# Molecular and Biochemical Mechanisms of Elicitors in Pest Resistance

**DOI:** 10.3390/life12060844

**Published:** 2022-06-06

**Authors:** Saif ul Malook, Saiqa Maqbool, Muhammad Hafeez, Samantha Chandranath Karunarathna, Nakarin Suwannarach

**Affiliations:** 1 Research Center of Microbial Diversity and Sustainable Utilization, Chiang Mai University, Chiang Mai 50200, Thailand; 2 Shenzhen Branch, Guangdong Laboratory for Lingnan Modern Agriculture, Genome Analysis Laboratory of the Ministry of Agriculture, Agricultural Genomics Institute Shenzhen, Chinese Academy of Agricultural Sciences, Shenzhen 518100, China; 3 Department of Chemistry, University of Agriculture Faisalabad, Sub-Campus Burewala, Faisalabad 38000, Pakistan; saiqamaqboolchem@outlook.com; 4 State Key Laboratory of Rice Biology, Ministry of Agriculture, Key Laboratory of Molecular Biology of Crop Pathogens and Insects, Institute of Insect Sciences, Zhejiang University, Hangzhou 310058, China; 0621598@zju.edu.cn; 5 Center for Yunnan Plateau Biological Resources Protection and Utilization, College of Biological Resource and Food Engineering, Qujing Normal University, Qujing 655011, China; samantha@mail.kib.ac.cn

**Keywords:** herbivore-associated elicitors, pattern-recognition receptors, plant defense, secondary metabolites, signaling metabolites

## Abstract

Insect herbivores have a variety of life cycles and feeding habits, making them extremely diverse. With their host plants, they form close relationships and suppress their defense mechanisms. Molecular elicitors are the key bio-elements in the detection and recognition of attacking enemies in tissue consumption. Insect oral secretion, frass, and fluid of egg deposition contain biologically active molecules called herbivore-associated elicitors (HAEs) that are recognized by pattern-recognition receptors (PRRs). Many plants distinguish insect feeding from wounding by HAEs present in their oral secretions (OS) and induce local and/or systemic responses against arthropod feeding. PRRs perceive HAEs in the oral secretion of caterpillars in a species-specific manner to elicit exclusive defense responses. HAEs-PRRs interactions induce plant resistance by reprogramming plant metabolism and transcriptional machinery. Quantitative, timely, and coordinated plant response initiate early signaling events, including Ca^2+^, reactive oxygen species (ROS), and mitogen-activated protein kinases (MAPKs). However, in insect herbivory, little is known about the molecular basis of signal transduction and regulation of plant resistance. We discuss here how early signaling cascades converge into the accumulation of phytohormones that regulate downstream special metabolites against herbivores. In this review, we propose a hypothetical model of PPRs-HAEs-mediated-induced responses in plants and discuss how PRRs-HAEs interactions elicit short- and long-term induced defenses in plants. The understanding of PRRs-HAEs interactions will help to explore the fundamental molecular mechanisms of host manipulation and may generate prospects to develop novel pest-resistance strategies.

## 1. Introduction

**Figure 1:**
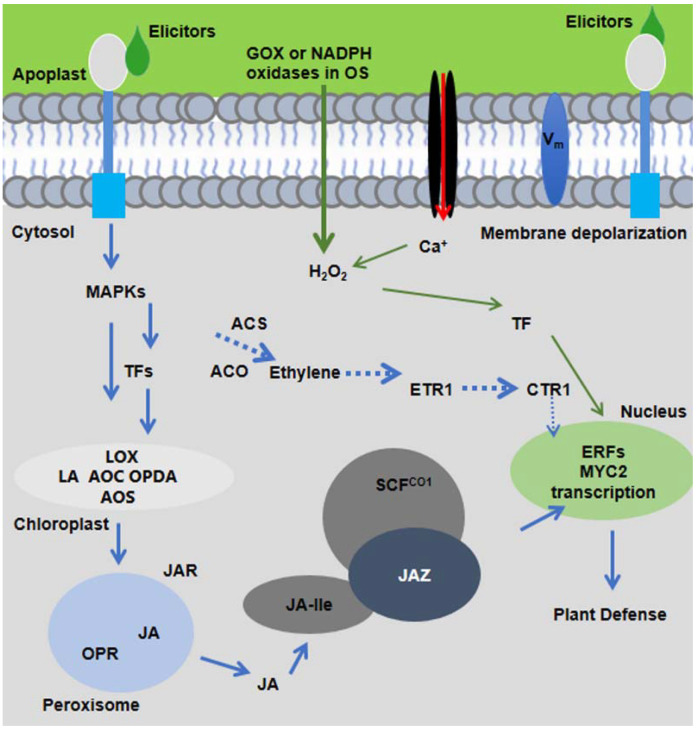
The molecular signaling model of plant response to insect herbivory. HAEs from oral secretion (OS) of insect herbivores are perceived by plant receptors present in plasma membrane. Within minutes of herbivore feeding, short signaling molecules, such as ROS, Ca^2+^, MAPK signaling, and membrane depolarization (*Vm*), are activated and elicit the JA-Ile production. JA-Ile binds with SCFCOI1 and triggers the degradation of JAZ and activates downstream plant defenses.

As sessile organisms, plants cannot escape from herbivore arthropods and are substantially challenged by insect herbivores. Over millions of years of coevolution with insects, plants have evolved exquisite defense mechanisms to fend off insect herbivory on plants [1]. The recognition of herbivore attacks requires the ability of plants to detect chemical cues (Herbivore-associated elicitors; HAEs) generated by insects during infestation, and these receptors are also called receptor kinases (RKs). Plants distinguish insect feeding from wounding by recognizing specific conserved molecules present in their oral secretions (OS; shown in Figure 1) [2,3,4,5]. In literature, based on plant-insect interactions, few reports have revealed that OS constituents depend on the insect feeding of host plants and their associated microbes [6].

During insect herbivory, deposition of OS on the wounds causes manipulation of plant responses against insect herbivores by changing plant metabolism and gene expression [4,7]. HAEs in nature are diverse in structure and exist in the form of enzymes [e.g., glucose oxidase (GOX) and *β*-glucosidase], lipids [fatty acid-amino acid conjugates (FACs) such as volicitin and caeliferins], cell wall fragments (e.g., pectin and oligogalacturonides), and plant peptides (e.g., inceptin: proteolytic fragments of the chloroplastic ATP synthase subunit), but none of them were found to affect the induced defenses of tomato [4,8,9,10]. The HAEs are not general elicitors in all plants, and plant responses to insect herbivores are restricted to plant-insect associations that depend upon the specific mode of feeding style of insects [7,11,12]. This specificity reflects the evolutionary history of both plants and insects living and surviving together in nature, and it is important to understand the mechanism of plant-elicitors interactions in an evolutionary context [4]. Herbivore-induced defenses are mediated by signaling molecules and are employed to maintain crop resilience during insect herbivory [12,13,14]. Thus, despite the need for a clear understanding of induced responses, plant receptor interactions with their HAEs remain an emerging research topic in plant-insect interaction.

Upon the recognition of insect elicitors, plants activate defense responses by triggering calcium ion influx (Ca^2+^), plasma membrane depolarization (*Vm*), mitogen-activated protein kinases (MAPKs), NADPH oxidase, production of reactive oxygen species (ROS), and activation of nitrogen species (NO) [15]. The molecular signaling cascades elicit the production of defense hormones, mainly jasmonic acid (JA), ethylene, and salicylic acid (SA) as well as transcription factors (TFs). Defense hormone, especially JA, is the central component to regulate downstream defense metabolites including but not limited to glucosinolates, benzoxazinoids (Bxs), cyanogenic glucosides, alkaloids, phenolics, and proteinase inhibitors in damaged and systemic leaves, as shown in Figure 1 [13]. Several excellent reviews have been published on the discussion of herbivore-induced plant responses [13,16,17,18]. Molecular breeding for pest resistance traits is of great importance in developing crops with enhanced insect resistance. To develop pesticide-free food for an increasing world population, a clear understanding of the underlying mechanism in the perception of crop attack by insect herbivores and how plant receptors are employed to induce plant defense responses against herbivory is needed. The prime objective of this review is to provide cutting-edge research updates about plant receptor interaction with HAEs and the perception of insect herbivory to induce signal transduction-mediated defense responses accordingly.

## 2. Plant Receptors Perceive Insect Herbivory

Despite the continuous battle for the survival between plants and insects, the evolution of plant defenses is the main weapon that has a decisive power to determine victory in favor of plants or insects. Molecular and chemical ecologists mainly focused on investigating HAEs in the last two decades, but information on HAEs interactions with PRRs is still at an emerging stage. The first step in the mechanism of HAEs recognition is the detection of HAEs in the oral secretion (OS) of herbivorous insects, which is mediated by membrane-bound receptors [19,20]. However, many receptors of HAEs were discovered, and we have listed the known receptors and their respective HAEs in Table 1. The weapons of plant perception are divided into receptor-like kinases (RLKs) or proteins with ecto- and cytosolic domains in the plasma membrane that are actively involved in ligand binding. Plasma membrane-localized, *leucine-rich repeat receptor-like kinases* (*LRR-RLK*) genes belong to the subfamily of receptor-like kinases (RLKs). The role of *RLKs* is largely known for the recognition of insect herbivory when OS elicitors bind with the plasma membrane in plants. For example, biological active tritiated volicitin [(^3^H)-L-volicitin) binds to the plasma membrane in maize, and this binding was increased by foliar application of methyl jasmonate (MeJA). Similarly, actual feeding by *Spodoptera exigua* enhanced four-folds binding capacity of volicitin with the plasma membrane, suggesting that volicitin binding with plasma membrane involves FAC-specific receptors. The receptors perceive MeJA and insect herbivory and activate gene transcription-encoding protein-binding with the plasma membrane, which depends on unknown receptors and JA signaling [21]. Our understanding of the perception of MeJA and volicitin binding with plasma membrane is not yet clear and needs further investigations. The *Bph3*, a cluster of three genes identified in rice, is known as G-type lectin receptor kinases (OsLecRK1-OsLecRK3) involved in resistance to brown planthopper and white-back planthopper. Molecular cloning and introgression of *Bph3* into susceptible rice cultivars lines demonstrated increased resistance to brown planthopper [22]. Leucine-rich repeat receptor kinases (LRR-RK) has been demonstrated to be involved in perception and resistance against *Chilo suppressalis* (striped stem borer; SSB). Feeding by SSB and treatment with OS of *Spodoptera frugiperda* increased expression level of *OsLRR-RLK1*, JA, SA, ethylene biosynthesis genes, and activity of trypsin protease inhibitor (*TrypPI*). The RNAi-mediated silencing of plants for *OsLRR-RLK1* expression showed attenuated activity of *TrypPI* and resistance to stem borer. We hypothesize that *OsLRR-RLK1* may bind with HAEs in rice to induce plant responses [23]. *Nicotiana attenuata*, a native tobacco plant to western North America, is well-equipped with defense strategies to overcome insect herbivory. Lectin receptor kinases (LecRK1) in tobacco functions in perceiving the elicitors in larval OS. FAC 18:3Glu was found in OS when *Manduca sexta* feeds on tobacco leaves. Silencing of *LecRK1* gene by virus-induced gene silencing (VIGS) and inverted repeated RNA interference revealed the susceptibility of *ir-LecRK1* plants to *M. sexta*. Larvae were grown better and gained higher weight on *ir-LecRK1* than wild-type plants, suggesting that *LecRK1* is essential to perceive and regulate plant responses to *M. sexta* [24].

Oviposition produces a necrotic zone at the egg-laying site, and the activity of egg deposition is perceived by LRKs to make the alert signal for plants to elicit defense responses. Mutant plants with a T-DNA insertion in the coding sequence of the *Lecrk-I.8* gene were detected to induce plant responses by egg extract. Treatment with egg extracts partially induce the relative *PR-1* expression and the level of *PR-1* expression was significantly increased in Col-0 plants, suggesting that *Lecrk-I.8* is the main receptor in perceiving the egg-derived elicitors in *Arabidopsis* [40]. It would be interesting to test whether *Lecrk-I.8* perceives other elicitors. 

The plant receptor-like kinase somatic embryogenesis receptor kinase 1 (SERK1) a distinct member of SERK family, is required for a nucleotide-binding, leucine-rich repeat protein (NB-LRR) called Mi-1 to function in conferring resistance against caterpillar larvae, including *Macrosiphum euphorbiae* (The potato aphid). Mutant tomato plants silenced with SlSERK1 expression exhibited a higher rate of survival of potato aphids than wild-type plants, where the aphids died during feeding, suggesting the important role of SlSERK1 in the perception of potato aphid attack [41]. Endogenous peptides are released into the apoplast upon wounding or insect feeding and are considered secondary danger signals. Systemin is an 18-amino-acid-long peptide molecule cleaved by prosystemin and spreads throughout a plant body to activate proteinase inhibitor that negatively affects the growth of caterpillars [29]. Many signaling events of plant resistance were initially described in tomatoes, including systemin induce responses, which are conserved in the plant kingdom. In *Arabidopsis*, *At*Pep peptide elicitors are recognized by two closely related leucine-rich repeat receptors, *AtPEPR1* and *AtPEPR2* [27,42,43]. In tomatoes, the high-affinity receptor *SYR1* of *LRR-RLK* family binds to systemin. Introgression lines that lack a single transgene *SYR1* were tested for *Spodoptera litoralis* resistance on tomatoes. Larvae of *S. littoralis* grow better and gain highly significant weight on introgression line *IL3-3*, which is deficient in *SYR1*, than wild-type tomato plants. However, the expression level of the proteinase inhibitor gene (*PIN1*) was not affected in local and systemic tissues of both transgenic lines, suggesting that *SYR1* triggers other defense molecules against *S. litoralis* larvae [29]. Exogenous treatment with ATP increased defense responses, including cytosolic Ca^+^ in *Arabidopsis*. ATP-insensitive mutant *DOES NOT RESPOND TO NUCLEOTIDE 1* (*DORN1*), which is defective in lectin receptor kinase I.9 (Lecrk-I.9), which binds with ATP and is required for ATP-induced responses. Overexpression of *DORN1* increased plant responses to wounding, indicating that *DORN1* is involved in the perception of extracellular ATP [28].

Recently, researchers in the Schmelz group used a forward genetic mapping approach to show that leucine-rich receptors in *Vigna unguiculata* (cowpea) and *Phaseolus vulgaris* (common bean) recognized proteolytic fragments of chloroplastic ATP synthase (inceptin) in OS and elicit the defense responses. Heterologous expression of leucine-rich repeat receptor in tobacco showed that inceptin receptor (INR) regulates plant defenses and confers resistance to *Spodoptera exigua* [44,45]. In contrast to the pathogen, receptors employed by plants to perceive insect herbivory are not well studied and need further investigations.

## 3. Detection of Herbivory and Encounter Mechanism

Plants have the ability to distinguish between insect herbivory and mechanical damage by the pattern of tissue feeding and are mediated by the perception of elicitor constituents present in the OS. For example, representative elicitors were chosen and applied to the wounds of different plant species to monitor the induction of phytohormones and volatiles. Results indicated that plants differently respond to insect-derived elicitors. suggesting that plant defense response to various insect herbivores is a species-specific phenomenon [46]. Metabolic study of fatty acid-amino acid conjugates (FACs), the main constituent in the OS of *M. sexta*, revealed that *N*-linolenoyle-glutamic acid (18:3-Gln) was metabolized within 30 s after applying to puncture wounds of *N. attenuata* leaves. Similarly, application of *M. sexta* OS into wounded leaves showed a 50% decline and changed into a modified form of 18:3 Gln that corresponded to 13-hydroxy-18:3-Glu, 13-hydroperoxy-18:3-Glu, and 13-oxo-13:2-Glu. *Nicotiana attenuata* silenced plants in the expression of *lipoxygenase 2* (*LOX2*) and *lipoxygenase 3* (*LOX3*), showing a strong reduction in the modified form of 18:3-Gln and suggesting that modified forms of FACs are responsible to elicit plant responses in *N. attenuata* [47].

Egg deposition of herbivores poses a severe threat to the survival of plants as they change into feeding caterpillars, and plants have developed necrotic arsenals at oviposition regions that are associated with high mortality and reduced hatching rate [48]. Oviposition by *Pieris brassicae* elicited an increased expression of hundreds of genes after egg deposition. Importantly, transcriptome signature by *P. brassicae* oviposition was strikingly different in *Arabidopsis* than observations drawn by chewing herbivores feeding, but oviposition shared similarities in defense-related gene expression changes induced by the biotrophic pathogen during infection [49]. Similarly, oviposition by *Pieris brassicae* elicited increased accumulation of salicylic acid (SA), and a similar response was observed after perception of pathogen-associated molecular patterns (PAMP). In addition, treatment with egg extract elicited a rapid and strong induction of PAMP-responsive genes, unraveling the shared plant receptors in insect oviposition and PAMP [40].

Frass deposition in a plant’s whorls can suppress plant defenses. FAW larvae deposit copious amounts of frass in whorls during foliar herbivory on maize plants. Applying fall armyworm frass extract to wounded leaves increased the performance of FAW larvae more than caterpillars grown on wounded plants, and frass treatment attenuated the transcript accumulation of defense-related genes, including JA, which indicates insects have encountered proteins that cheat/modulate the plant defenses [50]. During larval feeding on maize, frass can accumulate in maize whorls and deposit for a long period, damaging the plant tissue. Infestation by FAW induces maize chitinases *Pr4* and endochitinase A, and these chitinases deposit in the frass and mediate suppression of FAW-induced maize proteinase inhibitor, thereby increasing caterpillar growth in plants [51,52]. Natural enemies of pest herbivores become attracted upon emission of HIPVs, and maize specialist fall armyworm can suppress indirect defenses similar to direct defenses [11,53]. HIPVs emitted by fall armyworm infestation were much weaker than *S. littoralis*, *S. exigua*, and *Helicoverpa armigera* in maize, and FAW feeding could not suppress emission of HIPVs in *Gossypium herbaceum* (cotton), suggesting that HIPVs suppression is specific for maize [54].

## 4. Plant and Insect Origin Elicitors

Plants are exposed to biotic stresses by microbes, insects, and animal feeding. To fend off insect herbivory, plants have adapted responses and recognition systems that depend on specific HAEs. HAEs take part in signaling pathways and can activate the defense reaction system in plants. Apart from components of OS, HAEs originate in bacteria, caterpillar frass, the oviposition fluid, and some insect pheromone compounds that can either disrupt or induce plant defenses [55]. In other words, many molecules in OS can cause the plant to manipulate its defense response, involving enzymes such as glucose oxidase and *β*-glycosidase, peptides such as inceptin, and fatty acid conjugates such as volicitin (Table 1) [4,6,56]. However, as time passes, some plants can overcome this inhibition when they have adapted themselves to recognize the molecules from the insect [57]. Therefore, fatty acid-amino acid conjugates (FACs), or fatty acid amides, were one of the first types identified as an elicitor in the saliva of insects [10]. A two-pronged methodology to study FACs in *M. sexta* exhibited increased indirect defense response in a host plant by the inducing the volatiles organic compounds (VOCs) and attracting the predators [58]. Since the initial discovery, other types of elicitors have been identified, with their specific molecular activity varying greatly between plant species [46]. Furthermore, inceptins and caeliferins in oral secretions activate insect defensive pathways [59]. Moreover, in previous studies, the induction of defense signaling has been reported in response to the presence of glucose oxidase (GOX) in insect saliva, for example, the *Proteinase Inhibitor 2* (*PIN2*) produced by the salivary component of *Ostrinia nubilalis* induces in maize and tomato [60,61]. However, some OS inhibit the defense pathway in plants. According to the literature, it has been observed in the larval stages of *S. littoralis* and *P. brassicae*, where salivary secretions inhibited defense to allow larvae to grow [62]. As a result, depending on which organism oversees the evolutionary process at that time, the plant or the insect, these molecules can either activate or repress plant defense responses, respectively.

## 5. Elicitors of Plants’ Intracellular Products

**Figure 2:**
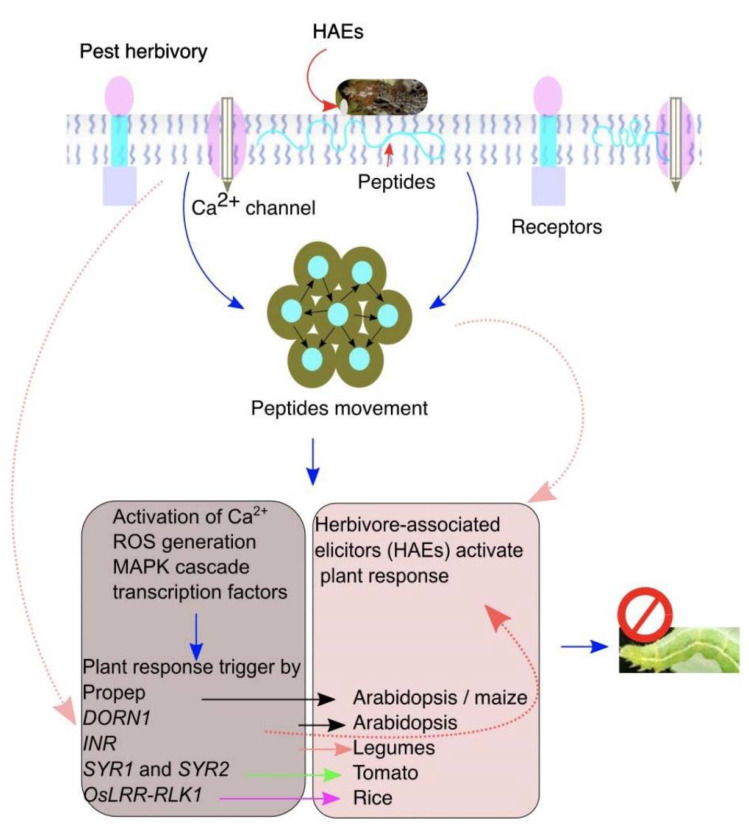
The plant pattern-recognition receptors (PRRs) recognize herbivore-associated elicitors (HAEs) to elicit plant responses against insect herbivory. A plant perceives and detects herbivory by recognizing HAEs on insect feeding. Damaged cells release intracellular molecules that move into the apoplast and to the undamaged neighboring cells to induce the plant responses. Insect feeding induces endogenous peptides that bind with HAEs to elicit the downstream plant defenses.

The simplest form of plant elicitors is the intracellular products released upon leaf damage by insect feeding [63]. The intracellular liquid moves to the apoplast and is recognized by DORN1/P2K1 in neighboring, undamaged cells and activates ATP-induced Ca^2+^ defenses (Figure 2 and Table 1) [28]. In tomatoes, degradation activity of adenosine-5-triphosphate (ATP) was detected in *Helicoverpa zea* OS assay with tomato leaf fluid. On the other hand, salivary glands of *H. zea* secrete apyrase and ATP-hydrolyzing enzymes that interfere with ATP signaling and suppress defense-related genes in tomatoes [64]. During insect feeding, regurgitation on leaves provides signaling cues recognized by plants. For example, lignocellulose deposited in the herbivore gut during feeding and digested products can be recognized by receptors in Arabidopsis upon e digestion, functioning as elicitors. However, it is not clear whether these compounds are gut-derived or are plant cell wall degradation products [2,65]. In maize and lima bean, insect feeding produces extracellular self-DNA (esDNA), and plants exposed to esDNA and extracellular heterologous DNA increased plasma membrane potential (*Vm*) and calcium flux (Ca^2+^), confirming that esDNA trigger plant responses [25]. Whether the perception of esDNA requires specific receptors other than insect herbivory is another interesting research question to answer.

## 6. Peptide Elicitors

Endogenous peptide molecules are secreted in plant cells and exclusively found in Solanaceae family and function to elicit plant defenses in response to herbivore feeding (Figure 2). In tomatoes, prosystemin is accumulated and processed in the cytosol by proteolytic cleavage and transported to the apoplast to trigger plant defenses by interacting with membrane-localized leucine-rich receptors (LRR) called PEPRs [66]. In *Arabidopsis*, endogenous peptide signals of *AtPep1* amplify the defense responses by *PEP receptor* (*PEPR1* and *PEPR2*) [67]. Systemin, a plant peptide hormone of 18 amino acids derived from prosystemin, is a larger precursor protein of about 200 amino acids. Systemin spreads throughout the plant upon wounding and negatively affects the growth of chewing herbivores by activating JA-dependent defenses. Genetic analysis revealed that systemin produces or enlarges the production of systemic signals. *Spodoptera lituralis* induced a greater level of transcript accumulation of *PEPR1*, *PEPR2*, and *PROSPEP3* in *Arabidopsis*. Genetic evidence by using *pepr1* and *pepr2* mutant plants showed that larvae grew much bigger on mutant plants than wild-types [68], suggesting that *PEPR1* and *PEPR2* play an important role in the perception of insect herbivory. In addition, systemic trigger the emission of green leaf volatiles (GLVs) to attract the natural enemies of caterpillars as a part of the role in tri-trophic interactions as well as direct defenses by increasing the accumulation of reactive oxygen species (ROS) and phytohormones [69,70]. Peptide elicitors (Pep), similar to systemin, are recognized in *Arabidopsis* (*AtPep1–8*) and maize (*ZmPep1,3*). In maize, *ZmPep3* induced an increased level of JA, ethylene, proteinase inhibitors, production of volatiles, benzoxazinoids, and genes encoding defense proteins. The induced plant responses by *zmPep3* were similar to those elicited by *S. exigu* [26]. In rice, brown planthopper infestation significantly induced transcript levels of both OsPep receptors and Pep precursors. Knockout mutant plants impaired in function of OsPEPRs demonstrated susceptibility to brown planthopper feeding, whereas exogenous application of OsPep3 improved the resistance level in rice seedlings against brown planthopper infestation as well as fungal pathogen *Magnaporthe oryzae* and bacterial pathogen *Xanthamonas oryzae* pv. *oryzae* [71]. It is clearly demonstrated that the OsPEPRs signaling is essential for plants to defend against insect herbivores.

## 7. Elicitors in OS of Insects

Among well-known HAEs, fatty acid–amino acid conjugates (FACs) are a best-studied group of elicitors, which trigger defense responses upon herbivore feeding in many plant species, including maize, soybean, eggplant, and tobacco [4,10,46]. A maize elicitor, volicitin, a hydroxyl FAC [*N*-(17-hydroxylinolenoyl)-L-glutamine], was isolated from the OS of *S. exigua* larvae by Alborn and his colleagues in 1997. Applying the volicitin onto the wounds of maize leaves elicits the emission of an increased level of volatiles and attracts parasitic wasps, natural enemies of *S. exigua*. Wounding without the application of volicitin did not emit the blend of volatiles that attract the natural enemies of herbivores [10]. Since the discovery of volicitin, several FACs have been found in OS of lepidopteran species, and their biological functions are well studied in *N. attenuate*. *Manduca sexta* larvae induced increased accumulation of MAPKs, JA, ethylene biosynthesis genes, metabolome, and transcriptome reprogramming in infested and systemic leaves [72,73,74,75,76,77]. Wounding elicits the increased transcript level of transcription factor (TF) WRKY3, and applying the FACs into the wounds of nicotiana leaves caused increased WRKY6 transcript accumulation. Importantly, WRKY3 is required for the elicitation of WRKY6, and silencing of either gene made plants susceptible to herbivores [75]. *Manduca sexta* herbivory induced high levels of salicylic acid-induced protein kinase (SIPK) and wound-induced protein kinase (WIPK). Silencing of *SIPK* and *WIPK* showed decreased defense levels, but *M. sexta* larvae grown on transgenic plants did not show a difference compared to wild-type plants.

However, green leaf volatiles (GLVs) were attenuated in both transgenic plants and wild-type plants, and the addition of synthetic GLVs restore the increased *M. sexta* performance in transgenic plants [74]. Glucose oxidase (GOX) is the main component of OS in *H. zea*, which acts as a salivary protein to suppress the herbivore-induced plant responses in *Nicotiana tabacum* (tobacco) [36]. In response to larval herbivory, *Medicago trancatula* (alfalfa) responds by saponins and terpenoid production. *Spodoptera exigua* (Beat armyworm) suppressed the transcript accumulation of saponins biosynthetic genes for terpenoid production. Researchers hypothesized that GOX may involve in the suppression of gene expression following insect feeding. Further experiments by comparing wounding, the addition of GOX into wounds, and insect feeding confirmed the function of GOX in suppressing defense responses in herbivore attacks [78].

Chemical analysis of the OS in *S. exigua* indicated that FACs are composed of fatty acid (linoleic acid (LA)/linolenic acid), which is plant originated and insect-derived amino acid (Glu/Gln). Interestingly, 17 hydroxylation and conjugation occur in the midgut of insects, which is important for the biological activity that emits plant volatiles to attract natural enemies of herbivores [79]. Feeding experiments with radio-labeled glutamine, glutamic acid, and linolenic acid to *S. litura* caterpillars revealed that FACs are involved in nitrogen assimilation and function as glutamine storage in the insect. Glutamine is the main component in insect nitrogen metabolism, and hence, it is not possible for caterpillars to stop production of FACs when feeding on plants, even though plants perceive caterpillar feeding in the presence of FACs [80]. 

In addition to FAC, there are several other elicitors reported in insect OS. Inceptin, a proteolytic fragment of the chloroplastic ATP synthase γ-subunit, an elicitor isolated in OS of *S. frugiperda*, perceives the insect herbivory and enhances production of ethylene as well as increases accumulation of phenylpropanoid, VOCs, and protease inhibitor in *Vigna unguiculata* (cowpea) [9]. Comparing treatment with FAW, OS of *Anticarsia gemmatalis*, a legume specialist herbivore (Velvetbean caterpillar; VBC), did not induce large production of ethylene and direct herbivory to induce a smaller level of predominant volatile (*E*)-4,8-dimethyl-1,3,7-nonatriene (DMNT). The examination of OS in VBC for the discovery of truncated form of inceptin in VBC suggested that truncated form of inceptin may be recognized by PPRs and thus suppress the plant defense [81].

## 8. Elicitors in OS of Non-Lepidopteran Insects

A new class of non-lepidopteran elicitors called “caeliferins” was isolated in OS of *Schistocerca americana* (grasshopper). FACs found in non-lepidopteran species, for instance, Yoshinaga and her colleagues identified FACs in the gut of two closely related cricket species, *Teleogryllus taiwanemma* and *T. Emma*, and larvae of fruit fly, with a similar composition of *N*-linolenoyl-L-glutamic acid and *N*-linoleoyl-L-glutamic [82]. Maize seedlings treated with OS of grasshopper emit a blend of volatiles similar to herbivores. Treatment with 5 μL OS of grasshopper emitted a blend of volatiles equal to emission by seedling treated with 100 pmol of volicitin [31]. 

In the event of coevolution, plants have developed a sophisticated system for the perception of herbivorous feeding through cues derived from insect feeding, saliva, OS, eggs, volatiles, and microbes [13,48,83]. In rice, *Nilaparvata lugens*-secreted mucin-like protein (NlMLP) was identified by transcriptome and proteome analyses. NlMLP was highly expressed in salivary glands and secreted into rice tissues during brown planthopper feeding. As an elicitor, NlMLP induced the expression of pathogen-responsive genes and increased level of callose deposition, suggesting the important role as an elicitor in rice-insect interactions [38]. In *Pisum sativum* (pea), oviposition by *Bruchus pisorum* (pea weevil) increased cell division and created tumor-like growth (neoplasm) at the egg-laying site. Neoplasm delays the entry of pea weevil larvae into pods, and this resistance is mediated by bruchins and can cause neoplastic growth by application of 0.5 pg into the pods [35].

## 9. Plant-Induced Responses

In response to insect feeding, plants trigger a cascade of short signaling molecules and production of defense-related phytohormones as shown in Figure 2 [13]. Within minutes of tissue damage in *Phaseolus lunatus* (lima bean), *Spodoptera littoralis* elicited membrane depolarization and Ca^2+^ influx. The membrane depolarization was high at the site of *S. littoralis* feeding and was decreased as the distance increased from the wound site [84]. Interestingly, a research report revealed that herbivore feeding and mechanical wounding induces membrane depolarization at long distances from damaged tissues, and membrane depolarization was dependent on glutamate receptor-like (GRL) channels. In coordination with electric signals, ROS signaling plays an important role in plant response to chewing insects. For instance, this long-distance wound-induced signaling system requires increased production of ROS by membrane-bound RBOHD protein [85]. The superoxide (O_2_^−^) generates NADPH oxidases, which are considered important pathogen defense, and the transcript level of *Narboh D* homologe in *N. attenuate* was increased by wounding and further amplified by *S. littoralis*. Consistently, *rbohD*-silenced plants were susceptible to *S. littoralis* [86]. Aphids induced a strong accumulation of ROS 3 h after feeding on a resistant near-isogenic line but not in a susceptible line. ROS accumulation was increased with the increase of NADPH oxidase activity only in resistant cultivars, suggesting the involvement of H_2_O_2_ in oxidase activity. In addition, insect feeding can rapidly activate the MAPK signaling cascade, which is a highly conserved signaling mechanism among all eukaryotes [76]. In rice, *OsMPK3* has been found to positively regulate the defense response against rice striped stem borer (SSB, *C. suppressalis*) by modulating JA biosynthesis [87].

Early signaling of membrane depolarization, ROS, and MAPK cascade converge into the accumulation of phytohormones JA and conjugate with amino acid isoleucine (Ile). JA-Ile binds with its receptor complex consisting of CORONATINE-INSENSITIVE1 (COI1) and JASMONATE-ZIM DOMAIN (JAZ). The interaction of JA-Ile and its receptor complex leads to the degradation of JAZ and releases transcription factors (TFs) constitutively suppressed by JAZ, activating the expression of downstream defense genes against insect herbivores [13]. Therefore, an early signaling mechanism could be essential for all aspects of plant defense and lead to the induction of plant defense downstream in the form of secondary metabolites and toxic proteins to herbivores. The early signaling events ensure the quantitative, coordinated, spatial, and temporal defense responses. Further research is required to identify and characterize receptors that perceive chemical compounds to elicit defense pathways in plants.

## 10. Plant Defense against Gall-Inducing Insects

Galls are induced on plants by viruses, mycoplasma, bacteria, fungi, nematodes, insects, mites, and other plants. They are defined by an abnormal plant organ development with ectopic cell proliferation and expansion, generating a wide range of gall morphologies [88,89]. Among them, insect-induced galls have attracted the attention of many researchers because of their unique shapes and wide range of variation. The estimated number of gall-inducing insects ranges from 21,000 to 211,000 [90]. Furthermore, host plant species span numerous phylogenetic lineages, suggesting that gall-inducing systems have evolved independently during the insect evolution [91,92]. Insect galls can be induced on plant leaves, stems, floral buds, flowers, fruits, or roots and exhibit unique shapes [93,94]. Gall-inducing effector candidates have been identified from the transcriptome analysis of ovaries and venom glands of two cynipid gall wasps, *Biorhiza pallida* and *Diplolepis rosae*, inducing galls on oak and rose, respectively [95], or the analysis of salivary gland proteome of root-galling grape phylloxera, *Daktulosphaira vitifoliae* [96]. However, there is no direct evidence showing that these effector candidates have gall-inducing activity in their host plants.

The host plants produce tannins for the protection and gall-inducing insects from herbivores. Aphid galls on *R. chinensis* accumulate gallotannin, and genes involved in gallotannin biosynthesis [97], gallic acid synthesis [98], and lignin biosynthesis [99] have been identified. In the developing gall of the chestnut gall wasp, *Dryocosmus kuriphilus*, on the Chinese chestnut, *Castanea mollissima*, the expression of genes related to metabolic processes, such as phenylpropanoid biosynthesis, secondary metabolism, and plant–pathogen interactions, was altered compared to that of non-infested leaves [100]. Galls induced on elm leaves by a gall-inducing aphid, *Tetraneura akinire*, were shown to express the genes encoding lignocellulose synthase, suggesting the reinforcement of cell walls to improve resistance to damage by aphids [101].

## 11. Regulation of Plant Responses at Primed Stage

Priming response is well-documented in plant-pathogen interaction [102]. In the last decade, studies have documented the phenomenon of priming that is triggered by HIPVs, egg deposition, insect herbivory BABA, systemin, and cytokinin to explain enhanced defense plant responses [103,104]. For example, perception of indole signal primes neighboring plants for enhanced release of herbivore-induced plant volatiles (HIPVs) as well as early defense signaling genes within a plant [105,106]. Similarly, HIPVs released upon *Mythimna separata* infestation (*Zea mays* L. cv. Royal Dent) primes the maize resistance to insect herbivory and leads to increasing the relative transcript levels of *Bowman-Birk type trypsin inhibitor* (*TI*) for five days, and in a promoter region of TI, a set of methylation sites were found demethylated [107]. Recently, *M. separata* herbivory direct feeding was shown to prime the maize defenses and showed elevated accumulation of benzoxazinoids, JA/JA-Ile, as well as an increased level of defense-related transcripts to *M. separata* feeding in maize systemic leaves [108]. *Diabrotica virgifera* infestation on roots of maize seedlings increased DIMBOA content in leaves, and the leaves were primed for the accumulation of chlorogenic acid after subsequent infestation by *S. littoralis* [109]. However, priming is not only limited to VOCs and direct insect feeding, but the exposure of plants with oviposition equally primes plant defenses and exhibits elevated resistance to insect herbivores. For example, oviposition by the lepidopteran generalist insect *S. exigua* causes higher mortality, retarded development, and inflicted less feeding damage on oviposition-experienced than on oviposition-unexperienced *Nicotiana attenuata* plants. In addition, oviposited plants showed a stronger induction of caffeoylputrescine (CP) and trypsin protease inhibitors (TPIs) [110]. A cross-resistance experiment showed that *S. exigua* larvae suffered reduced performance on *M. sexta*-oviposited. *attenuata* plants as they did on *S. exigua-*oviposited plants [102]. Insect feeding by *Pieris rapae* and *S. exigua* in the previous generation on Arabidopsis plants primed enhanced resistance to *P. rapae* caterpillars in the next generation. Arabidopsis mutants deficient in JA perception or biogenesis of small interfering RNAs were not able to show inherited resistance, suggesting that JA signaling and epigenetics are likely to be involved in transgenerational priming [111].

**Figure 3:**
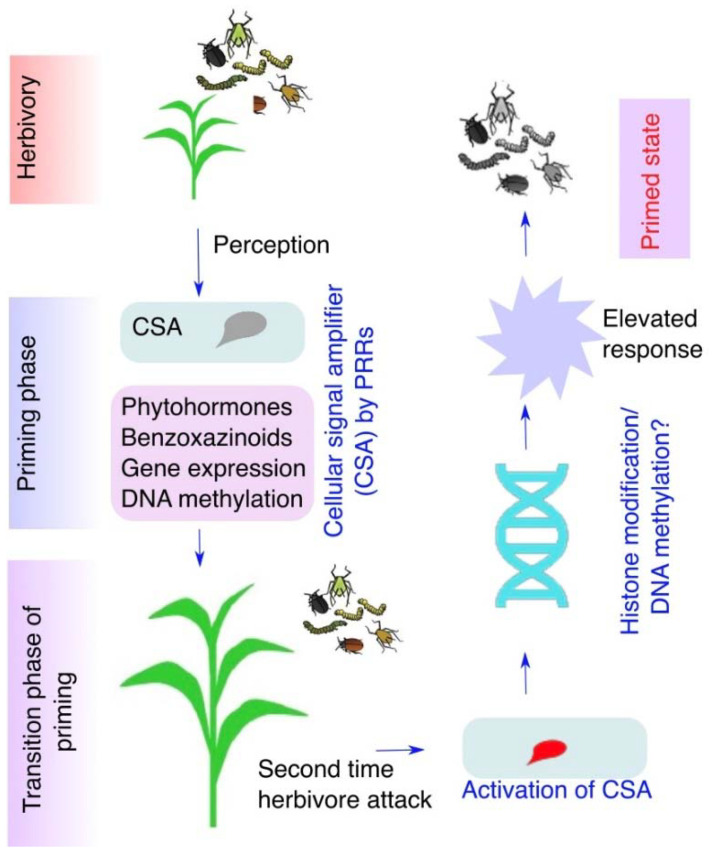
Caterpillar feeding activates the cellular signal amplifier (CSA) and primes plant responses for enhanced resistance. Caterpillars secrete oral secretion during feeding on plant leaves. Plant pattern-recognition receptors on the surface of plasma membrane specifically perceive the elicitors in the OS and trigger the activation of the inactive cellular signal amplifier (CSA). In response to herbivory CSA enhances the defense response by jasmonic acid (JA), benzoxazinoids (Bxs), gene expression, and DNA methylation to herbivorous feeding in plants.

Although growing lines of evidence have been implicated in the mechanism of priming with epigenetic-based histone modifications and DNA methylation, these epigenetic modifications are responsible for the changes in gene expression of defense-related genes that enable priming response stronger and faster in plants [112]. The hypothetical model of priming phenomena in maize induced by lepidopteran insects has been demonstrated (Figure 3). The mechanism of priming and involvement of epigenetic regulation is at a premature stage, and it would be worthy to investigate how epigenetic modifications regulate priming responses in plants. However, the mechanism of priming induced by insect herbivory not only in local but also in systemic, undamaged leaves has not been investigated so far and deserves much attention.

## 12. Conclusions

### 12.1. Concluding Remarks

- Although the mechanism of induced responses is important to understand for better protection of plants, recently, the debate on how plants perceive HAEs to activate downstream-induced defenses has received great attention. However, investigations are required to explore the receptors that perceive insect herbivory.
- Plant receptors perceive elicitors from endo- and exogenous danger signals that are both plant and insect-derived to activate the long- and short-term downstream defenses.
- Upon the perception of herbivory, plants can respond by using exquisite defense strategies. As the perception is strong, plant responses are more robust against caterpillars. The potential of plants to recognize and distinguish between mechanical damage and the kind of insect herbivory indicate the capability of perception of the chemical cues present in the OS of attacker herbivores and feeding on specific host plants.
- Plant responses to insect herbivory are very specific according to the HAEs, and the specificity of plants largely depend on the perception of the nature of elicitors.
- As plants respond stronger and faster to repeated herbivore attacks, it would be interesting to know whether, how, and to what extent plant receptors are involved in the induction of long-term responses in plants.

### 12.2. Outstanding Questions

- To date, molecular signaling and biosynthesis mechanism of HAEs, such as FACs, caeliferins, egg deposition, and frass, to elicit the defense responses have not been extensively studied. It would be noteworthy to investigate the molecular signal transduction mechanism and biosynthesis of HAEs in plants and insects. Genome editing (e.g., knockout lines) and comparative transcriptomic approaches could be used for functional characterization.
- Indirect defenses are major shareholders in the repellence of herbivores. Genetic and functional characterization are required for the demonstration of genetic control of indirect responses and whether receptors perceiving insect herbivory could also function to emit volatiles to attract natural enemies to fend off insect herbivory.
- Insect infestation triggers short- and long-term plant responses. Defense is costly. There is a need to investigate how plant-defined long-term defenses are sustainable. Studies designed on the hypothesis of trade-off mechanisms could explain long-term and short-term sustainable defense responses.
- The molecular mechanism of induction of defense priming is still at immature stage. According to the specificity of plant responses to insect herbivory, it would be interesting to identify the HAEs that elicit plant responses and prime for enhanced resistance.
- Plants are constantly facing threats to their survival. To what extent plants manage resources for growth and defense by employing the receptors of pathogens and insects requires further research.

## Figures and Tables

**Table 1:** The list of HAEs and their known receptors against insect herbivory.

Elicitors	Receptors	Source of Elicitors	Host Plant	References
DNA	n.d.	These elicitors are of plant source	Bean, maize	[25]
Pep	Pep receptor (PEPR)		Maize	[26,27]
ATP	ATP receptors (DORN1/P2K1)		*Arabidopsis*	[28]
Systemin	Systemin receptor (SYR1)		Tomato	[29]
FACs (volicitin)	Unknown membrane proteins	*Spodoptera exigua*	Maize	[10]
*β*-Glucosidase	n.d.	*Pieris brassicae*	Maize	[30]
Caeliferins	n.d.	*Schistocerca americana*	Maize	[31]
Inceptin	Inceptin receptor (INR)	*Spodoptera frugiperda*	Maize	[9]
Lipase	n.d.	*Schistocerca gregaria*	*Arabidopsis*	[32]
Porin-like proteins	n.d.	*Spodoptera littoralis*	*Arabidopsis*	[33]
*β*-Galactofuranose polysaccharide	HAK/PBL27	*Spodoptera* spp.	*Arabidopsis*	[34]
Bruchins	n.d.	*Bruchus pisorum*, *Nilaparvata lugens*	Cowpea, pea	[35]
Glucose oxidase	n.d.	*Helicoverpa zea*, *Spodoptera exigua*, *Helicoverpa armigera*	*Nicotiana*	[36,37]
Mucin-like protein	n.d.	*Callosobruchus maculatus*	Rice	[38]
Oligouronides	n.d.	Produced by breakdown of plant cell walls by insect feeding	Tomato	[39]

n.d. = not detected.

## Data Availability

Not applicable.
